# Architecting Hierarchical WO_3_ Agglomerates Assembled With Straight and Parallel Aligned Nanoribbons Enabling High Capacity and Robust Stability of Lithium Storage

**DOI:** 10.3389/fchem.2021.834418

**Published:** 2022-02-02

**Authors:** Xiaotong Dong, Yongshuai Liu, Shikai Zhu, Yike Ou, Xiaoyu Zhang, Wenhao Lan, Haotian Guo, Cunliang Zhang, Zhaoguo Liu, Shuai Ju, Yuan Miao, Yongcheng Zhang, Hongsen Li

**Affiliations:** ^1^ Center for Marine Observation and Communications, College of Physics, Qingdao University, Qingdao, China; ^2^ School of Chemistry and Chemical Engineering, Henan Engineering Center of New Energy Battery Materials, Henan Key Laboratory of Bimolecular Reorganization and Sensing, Shangqiu Normal University, Shangqiu, China

**Keywords:** WO_3_, hierarchical structure, nanoribbons, lithium-ion batteries, high performances

## Abstract

The pursuit of electrochemical energy storage has led to a pressing need on materials with high capacities and energy densities; however, further progress is plagued by the restrictive capacity (372 mAh g^−1^) of conventional graphite materials. Tungsten trioxide (WO_3_)-based anodes feature high theoretical capacity (693 mAh g^−1^), suitable potential, and affordable cost, arousing ever-increasing attention and intense efforts. Nonetheless, developing high-performance WO_3_ electrodes that accommodate lithium ions remains a daunting challenge on account of sluggish kinetics characteristics and large volume strain. Herein, the well-designed hierarchical WO_3_ agglomerates assembled with straight and parallel aligned nanoribbons are fabricated and evaluated as an anode of lithium-ion batteries (LIBs), which exhibits an ultra-high capacity and excellent rate capability. At a current density of 1,000 mA g^−1^, a reversible capacity as high as 522.7 mAh g^−1^ can be maintained after 800 cycles, corresponding to a high capacity retention of ∼80%, demonstrating an exceptional long-durability cyclic performance. Furthermore, the mechanistic studies on the lithium storage processes of WO_3_ are probed, providing a foundation for further optimizations and rational designs. These results indicate that the well-designed hierarchical WO_3_ agglomerates display great potential for applications in the field of high-performance LIBs.

## Introduction

The commercialization of electrochemical energy storage (EES) systems, especially lithium-ion batteries (LIBs), has brought revolutionary changes in the industrial structure of energy storage (Abakumov et al., 2020; Liu L. et al., 2020; Eum et al., 2020; Gao et al., 2020). However, in spite of the mature technology of LIBs, which undergoes uncasting optimization for decades, the LIB systems are still seriously bottlenecked by conventional graphite materials on account of their low theoretical capacities, thus severely restricting the overall energy density of EES devices (Zhang and Qi, 2016; Qu et al., 2019). In this respect, considerable efforts were devoted to design and exploit novel alternative anode materials with larger reversible capacity and energy density (Li H. et al., 2021; Li Q. et al., 2021; Yue et al., 2022). As a renowned part of the potential electrode material family, tungsten trioxide (WO_3_) has attracted tremendous attention due to its excellent chemical stability, high theoretical capacity (693 mAh g^−1^), and low cost (Yao et al., 2017; Bekarevich et al., 2020).

Although WO_3_ has been widely investigated as hosts for energy storage, the applications of WO_3_ in LIBs are still hindered by its intrinsic inferior conductivity and large volume strain, resulting in poor rate capabilities, sluggish kinetics, and rapid capacity fading in practical applications, thus seriously inhibiting its further development (Sasidharan et al., 2012; Yoon et al., 2014; Bekarevich et al., 2020; Xiao Y. et al., 2021; Rastgoo-Deylami et al., 2021; Hou et al., 2022). To conquer the obstacles above, various strategies have been approached in recent reports, in which the well-known examples of one-dimension (1D) structure offered the natural starting point (Luo et al., 2021). 1D single crystal WO_3_ nanowires fabricated by Gu et al. delivered a discharge capacity of 218 mAh g^−1^ for the first cycle under a current of 50 mA g^−1^, and a capacity retention of 75.2% after 50 cycles, which can be largely attributed to the robust structural stability given by the 1D structure (Gu et al., 2007; Huang et al., 2020; Li et al., 2020). On the other hand, hierarchical structures have demonstrated their favorable Li-ion storage properties (Zhao et al., 2019; Liang et al., 2022). Duan et al. prepared biconical hexagonal (h-WO_3_) mesocrystals by an ionic liquid-assisted hydrothermal route; the specific capacity of h-WO_3_ mesocrystals can be maintained at 426 mAh g^−1^ at 50 mA g^−1^ after 50 cycles, benefiting from its inherent uniform porosity associated with well-defined nanoparticle orientation (Duan et al., 2015; Liu C. et al., 2020). However, noticeable capacity degradation was observed (1,379 mAh g^−1^ for initial discharge and 30.9% capacity retention on 50th cycle). Beyond these, vacancy engineering (Tu et al., 2018; Li et al., 2019) or developing hybridization of WO_3_ with conductive carbon-based materials (Yu et al., 2013; Di et al., 2019; Lee et al., 2021) have also been used to lower the bandgap and diffusion barriers, but the capacity retention is still limited during prolonged cycling. In this respect, despite these attempts, it remains an enormous challenge to improve the specific capacity and cyclic stability concurrently to satisfy future large-scale commercial applications (Xiao Z. et al., 2021; Du et al., 2021).

In light of the above consideration, herein, the well-designed WO_3_ agglomerates assembled with straight and parallel aligned nanoribbons are prepared *via* a one-step hydrothermal method for high-performance LIBs. Benefiting from the unique hierarchical structure of WO_3_ agglomerates with high electrode–electrolyte contact area, short Li-ion pathway, and good strain accommodation, the electrode exhibits dramatically enhanced Li-ion storage properties in cyclic stability, specific capacity concurrently, along with a high capacity retention of ∼80% (661.5 mAh g^−1^ for initial discharge and 522.7 mAh g^−1^ after 800 cycles at 1,000 mA g^−1^), remarkably higher than those of the state-of-the-art WO_3_-based anodes. Moreover, kinetics analysis and Li-ion diffusion chemistry of the hierarchical WO_3_ agglomerates are investigated, providing a deeper insight into the mechanical origins of the improvement on Li-ion storage properties. These intriguing findings showcase the intrinsic desirable Li-ion storage performance of the hierarchical WO_3_ agglomerates and open new opportunities for the applications of high-performance LIBs by informing more rational designs.

## Experimental Section

### Synthesis of Hierarchical WO_3_ Agglomerates

All the chemicals were used as received without further purifications. In a typical synthesis of hierarchical WO_3_ agglomerates, 0.3 g of (NH_4_)_10_W_12_O_41_·xH_2_O and 0.2 g of C_2_H_2_O_4_·2H_2_O dispersed in 30 ml of deionized water. After stirring for 30 min, the homogeneous solution was transferred into a 60-ml Teflon-lined autoclave and hydrothermal reaction proceeded at 180°C for 8 h in an electric oven. After cooling to room temperature, the obtained products were washed sequentially with deionized water and ethanol for three times, and they were finally dried overnight at 60°C. For comparison, pure WO_3_ with different morphologies were synthesized *via* the same route except that the hydrothermal time was set as 4 and 12 h, respectively.

### Material Characterization

The phase purity of the products was detected by powder X-ray diffraction (XRD, Bruker D8 Advance, Germany) with a Cu Kα radiation. X-ray photoelectron spectroscopy (XPS) measurements were performed by an ESCALAB250Xi system using a monochromatic Al Ka1 source. The surface morphology of materials was explored by field-emission scanning electron microscopy (FESEM, ZEISS, Sigma-300) and transmission electron microscopy (TEM, JEOL, JEM-2100F).

### Electrochemical Measurement

The working electrode slurries were obtained by mixing the active materials, Super P, and carboxyl methyl cellulose (CMC) (weight ratio = 7:2:1) in deionized water. The obtained slurries were coated on the copper foil; 1 M solution of LiPF_6_ in ethylene carbonate (EC)/dimethyl carbonate (DMC)/diethyl carbonate (DEC) with a volume ratio of 1:1:1 was employed as the electrolyte, and the Celgard 2,250 film (Whatman) was selected as separator. The galvanostatic discharge–charge measurements were performed on a multichannel battery tester (LAND-CT2001A). The cyclic voltammetry (CV) and electrochemical impedance spectroscopic (EIS) curves were conducted on an electrochemical workstation (IVIUM technologies, Vertex).

## Results and Discussion

**FIGURE 1:**
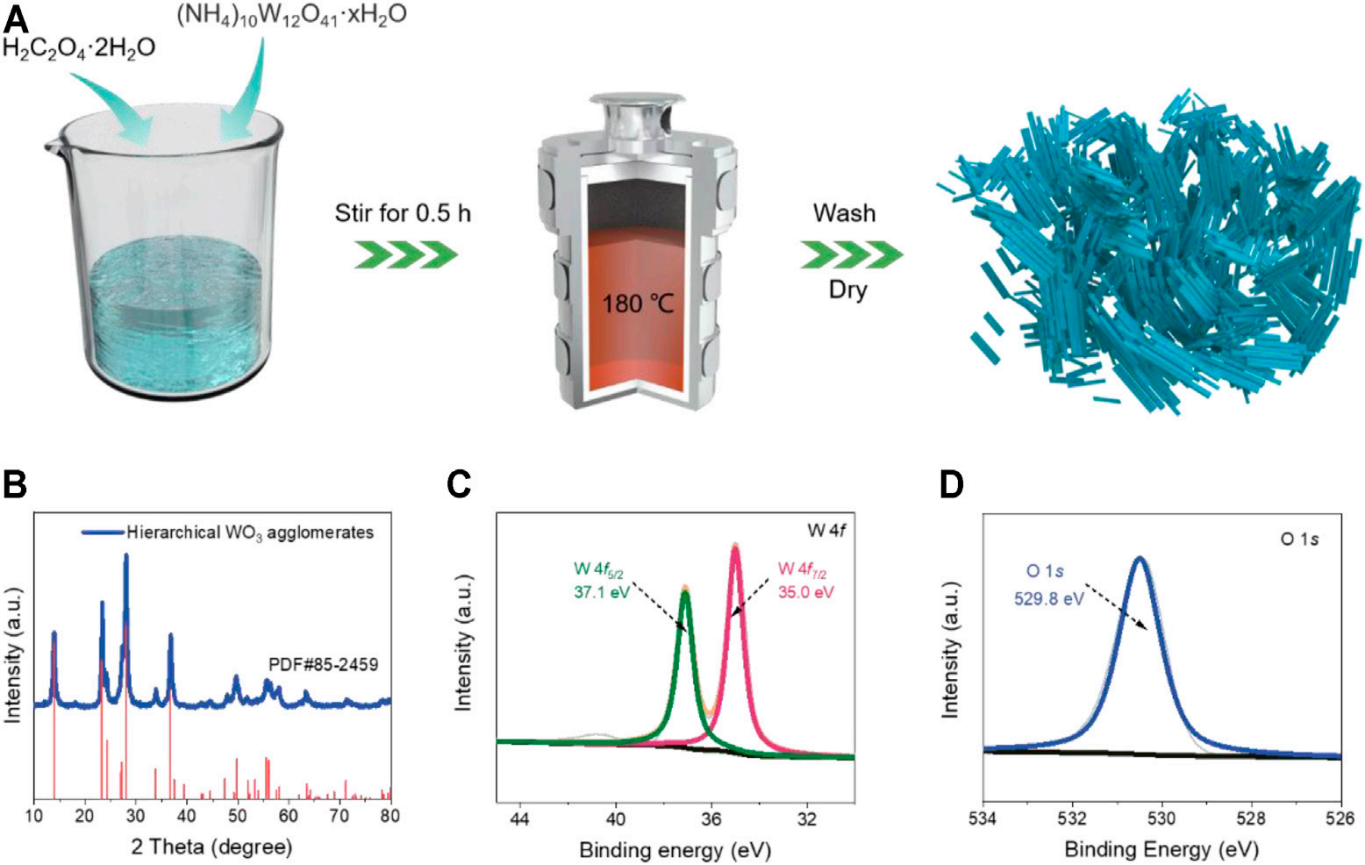
**(A)** Synthetic route of the prepared WO_3_ product. **(B)** The XRD patterns of the hierarchical WO_3_ agglomerates. **(C)** W 4*f* spectra and **(D)** O 1*s* spectra of the hierarchical WO_3_ agglomerates.

Figure 1A provides a schematic description of the synthesis strategy of the hierarchical WO_3_ agglomerates, which was prepared *via* a simple one-step hydrothermal method using (NH_4_)_10_W_12_O_41_·xH_2_O as the tungsten source (details are described in the Experimental section). Through regulating the hydrothermal time, the pure WO_3_ products with controllable morphology could be obtained. The XRD testing was first carried out to investigate the phase purity of the as-prepared products. As shown in Figure 1B, the green lines stand for the final products. The obvious sharp and strong diffraction peaks indicates high-crystallinity of WO_3_, which can be well indexed to typical hexagonal (JCPDS No. 85-2459) phase without any unknown impurity peaks (Pachfule et al., 2016). The XPS spectrum was also performed to analyze the elemental composition and surface chemical bonding state of the products (Tong et al., 2016). The survey spectrum shows that the prepared WO_3_ consists of C, O, and W elements (Supplementary Figure S1). The high-resolution XPS of W 4*f* (Figure 1C) exhibits two characteristic peaks located at 35.0 and 37.1 eV that correspond to the W 4*f*_7/2_ and W 4*f*_5/2_ levels (Grugeon et al., 2001). The O 1*s* XPS spectra in Figure 1D shows the peaks at 529.8 eV, which is ascribed to the W-O bonding mode of WO_3_ (Poizot et al., 2000). The XPS results further confirm the phase purity of WO_3_, in keeping with the above XRD analysis.

**FIGURE 2:**
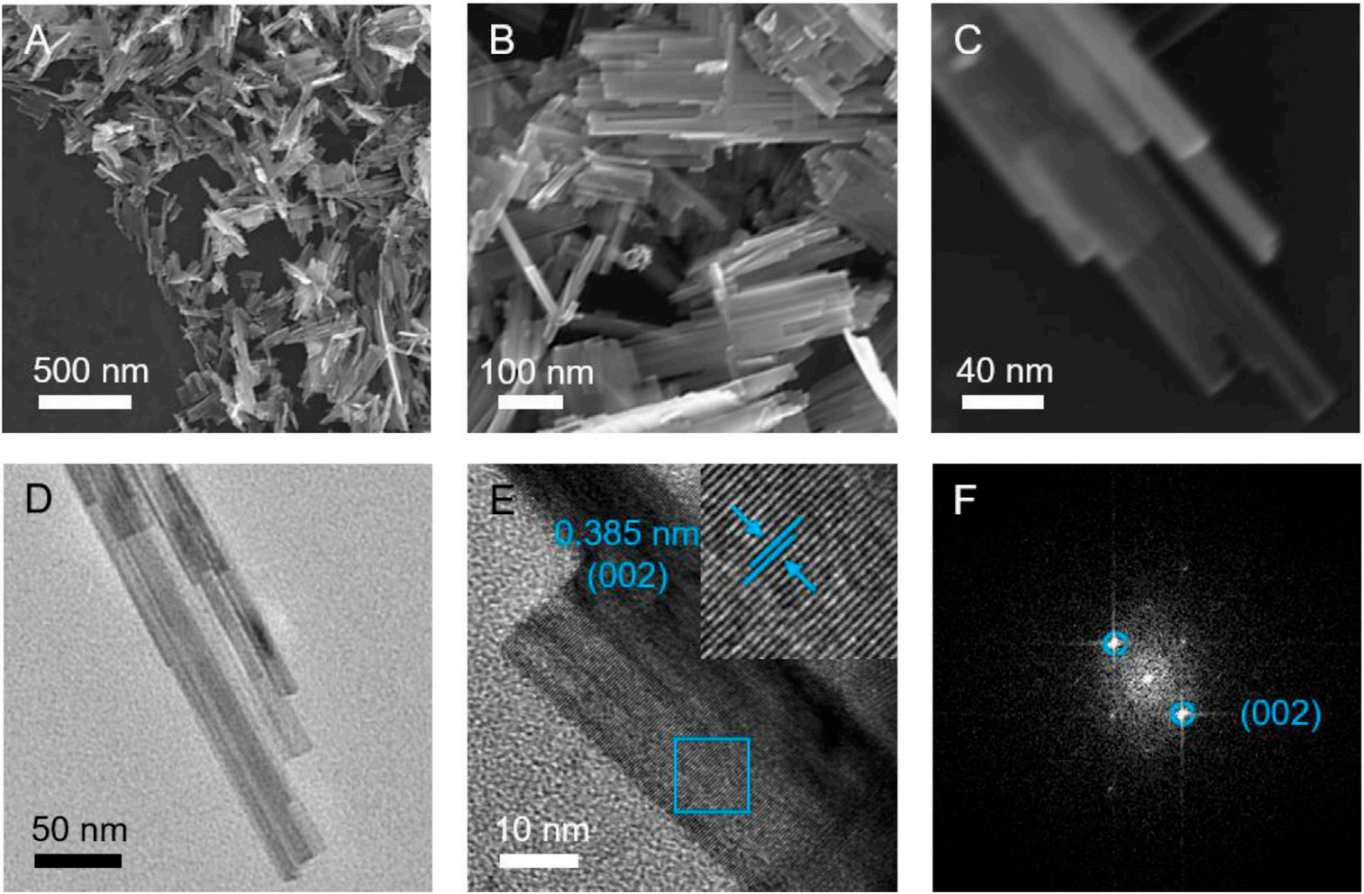
**(A–C)** FESEM, **(D)** TEM, and **(E)** HRTEM images of the hierarchical WO_3_ agglomerates assembled with straight and parallel aligned nanoribbons. **(F)** The corresponding FFT patterns.

To study the morphologies and microstructures of the prepared WO_3_ products, FESEM and TEM were conducted (Figure 2). As displayed in Figures 2A–C, the structure of product consists of straight and parallel aligned nanoribbons with a periodic stacking. These secondary 1D nanoribbons have a diameter of approximately 20 nm, offering abundant active sites for lithium ions/electrons exchange between the electrode and the electrolyte, (Liu L. et al., 2020), which is conducive to achieving good electrochemical performances. Supplementary Figure S2 and Supplementary Figure S3 exhibit the FESEM images of the contrast samples synthesized under different hydrothermal time. Interestingly, with the decrease of the hydrothermal time, the product features a multilayer brick morphology (Supplementary Figure S2, denoted as WO_3_ bricks); however, when the hydrothermal time increases, a micro-sphere assembled with some irregular nanoribbons can be obtained (Supplementary Figure S3, denoted as WO_3_ micro-spheres). It can be seen that the hydrothermal time plays a key role in the formation of the different structures of the WO_3_ electrode. In addition, the XRD patterns of the above-mentioned WO_3_ bricks and WO_3_ micro-spheres were also tested, which can be found in Supplementary Figure S4 and Supplementary Figure S5, respectively. TEM and high-resolution TEM (HRTEM) images of the hierarchical WO_3_ agglomerates (shown in Figures 2D,E) further reveal the crystallographic orientation and unique stacking straight and parallel aligned nanoribbons structure (Chen et al., 2019). The lattice-resolved HRTEM image exhibits the clear lattice with a spacing of 0.385 nm for the (002) planes of WO_3_ (Figure 2E). Moreover, the corresponding fast Fourier transform (FFT) pattern (Figure 2F) also shows the existence of (002) facets, further confirming the high purity of the prepared WO_3_.

**FIGURE 3:**
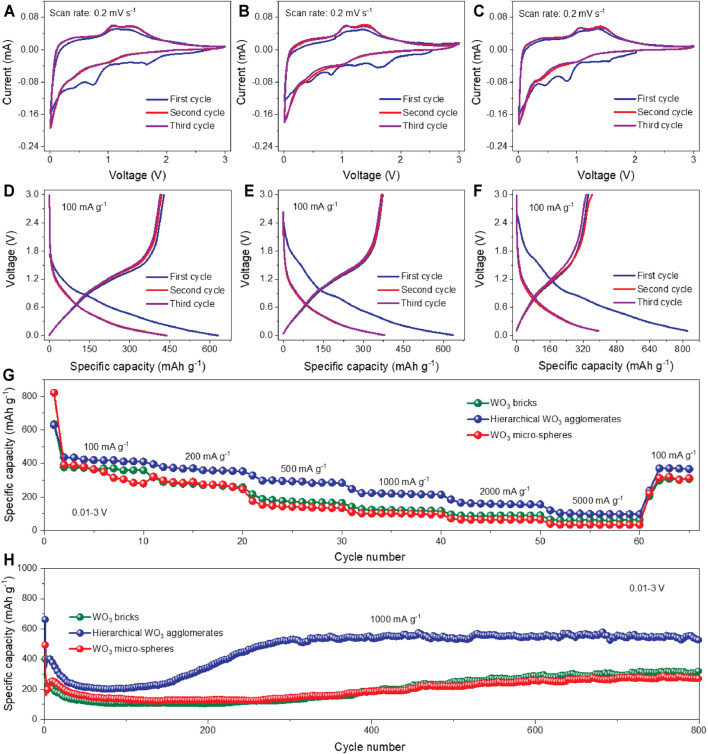
Electrochemical performance of the prepared WO_3_ products. **(A–C)** CV curves of the hierarchical WO_3_ agglomerates, WO_3_ bricks, and WO_3_ micro-spheres at a scan rate of 0.2 mV s^−1^, respectively. **(D–F)** Galvanostatic charge/discharge profiles of the hierarchical WO_3_ agglomerates, WO_3_ bricks, and WO_3_ micro-spheres at 100 mA g^−1^ during the first three cycles, respectively. **(G)** Rate performance of these prepared WO_3_ samples at various current densities from 100 to 5,000 mA g^−1^. **(H)** Cycling performances of these three prepared WO_3_ samples at 1,000 mA g^−1^.

To explore the potential of these as-prepared WO_3_ products for Li-ion storage, half-cells were assembled with Li metal as the counter and reference electrodes. The CV curves of the hierarchical WO_3_ agglomerates at a scan rate of 0.2 mV s^−1^ in the voltage window of 0.01–3.0 V (*vs.* Li/Li^+^) are presented in Figure 3A. Apparently, the reduction peaks between 0.3 and 2 V in the cathodic polarization process are observed in the first cycle, which can be ascribed to the decomposition of the electrolyte and the formation of the solid–electrolyte interface (SEI) (Dahbi et al., 2016; Wang et al., 2018; Yao et al., 2018; Huang et al., 2020). On the following cycles, the broad reduction peak at ∼1.0 V on the cathodic scan and the corresponding oxidation peak at ∼1.5 V on the reversible anodic scan are related to the Li^+^ intercalation–deintercalation processes (Yoon et al., 2014; Li B. et al., 2016; Pathak et al., 2018; Huang et al., 2020). Additionally, the reduction peak observed below 0.25 V can be associated with the conversion of W^6+^ to W^0^, which could cause the destruction of WO_3_ lattice structure to a certain extent (Rastgoo-Deylami et al., 2021). The CV curves of the pure WO_3_ bricks and WO_3_ micro-spheres are also shown in Figures 3B,C, which exhibits similar shapes to that of hierarchical WO_3_ agglomerates. For further comparison, Figures 3D–F illustrate the charge/discharge curves of hierarchical WO_3_ agglomerates, WO_3_ bricks, and WO_3_ micro-spheres under a stationary current density of 100 mA g^−1^ within the voltage range of 0.01–3.0 V (vs. Li/Li^+^). Note that, from all the three electrodes, the difference in discharge-specific capacity is quite obvious between the first two cycles, but achieves stability in the subsequent cycles, which can be possibly explained by the stable surface state and electrochemical reversibility after the initial activation process (Liang et al., 2021a; Wang et al., 2021). However, the hierarchical WO_3_ agglomerate electrode exhibits a higher discharge capacity of 487.6 mAh g^−1^ after the first cycle, much better than WO_3_ bricks (377.7 mAh g^−1^) and WO_3_ micro-spheres (393.6 mAh g^−1^). The superior performance of the hierarchical WO_3_ agglomerates was also observed in rate capability tests under various current densities, which is plotted in Figure 3G. A highly reversible capacity of 437.7 mAh g^−1^ at 100 mA g^−1^ is obtained, maintaining 121.4 mAh g^−1^ when the current density reaches as high as 5,000 mA g^−1^, demonstrating the best sustainable high current endurance of the hierarchical WO_3_ agglomerates among all the samples. Regarding the long-term cycling stability (Figure 3H), reversible capacities of 522.7, 325.9, and 272.3 mAh g^−1^ are retained for the hierarchical WO_3_ agglomerate, WO_3_ brick, and WO_3_ micro-sphere electrodes, respectively, after 800 cycles at a high current density of 1,000 mA g^−1^, indicating an outstanding long cycle life of the prepared hierarchical WO_3_ agglomerates. We further compared the performance parameter with state-of-the-art representatively reported congeneric LIBs, which are shown in Supplementary Figure S6 and Supplementary Table S1.

**FIGURE 4:**
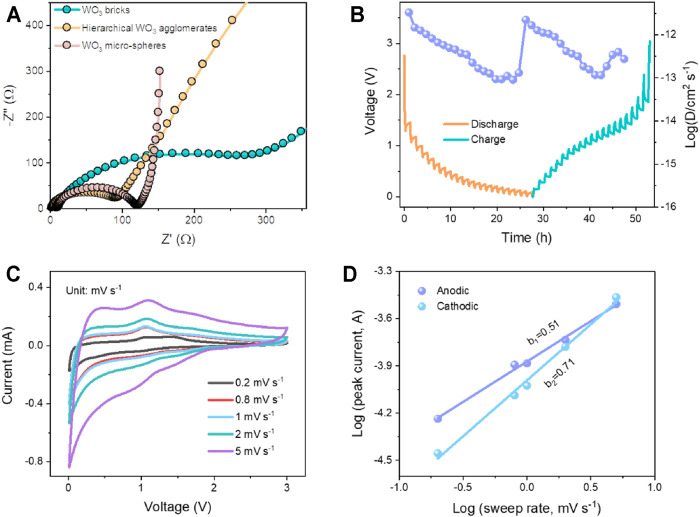
**(A)** Nyquist impedance plots for the hierarchical WO_3_ agglomerates, WO_3_ bricks, and WO_3_ micro-sphere electrodes, respectively. **(B)** GITT curves of the hierarchical WO_3_ agglomerates and the corresponding diffusion coefficients versus state of charge and discharge. **(C)** CV curves of the hierarchical WO_3_ agglomerate electrodes at different scan rates. **(D)** Corresponding plots of log(*i*) versus log(*v*) at cathodic and anodic peaks.

To study the dynamic characteristics of the hierarchical WO_3_ agglomerate, WO_3_ brick, and WO_3_ micro-sphere electrodes, the EIS measurement was carried out (see Figure 4A). The depressed semicircle in high-frequency regions represents the charge transfer resistance at the interface of electrode/electrolyte, while the inclined line in low-frequency regions associated with the mass transfer process (Shaibani et al., 2020; Jiang et al., 2021). Obviously, the EIS result reveals that the hierarchical WO_3_ agglomerate electrode shows significant smaller charge transfer resistance compared to that of WO_3_ brick and WO_3_ micro-sphere electrodes, which could be ascribed to the larger specific surface area and structural integrity of the hierarchical WO_3_ agglomerate electrode. To seek an in-depth understanding of the electrode kinetics of the hierarchical WO_3_ agglomerate electrode materials, the galvanostatic intermittent titration technique (GITT) is conducted to analyze the Li-ion diffusion coefficient (D_*Li+*_) during lithiation and delithiation. Of note, the cell was given five cycles at 100 mA g^−1^ to reach its thermal equilibrium state (Chen et al., 2019). In addition, the batteries alternately discharged for 15 min, rested for 60 min, and then charged for 15 min in the same way. D_Li+_ can be calculated *via*

$$D_{Li+}=\frac{4}{\pi \tau }{\left(\frac{mV_{m}}{MA}\right)}^{2}{\left(\frac{\Delta E_{s}}{\Delta E_{\tau }}\right)}^{2}$$ (1)

where τ represents the relaxation time, and m, M, V_m_, and A represent the active material mass, molar mass, molar volume, and the electrode geometric area, respectively. ΔE_s_ denotes the voltage changes during the relaxation period, and ΔE_τ_ is the variation during the current pulse. As shown in Figure 4B, the calculated D_*Li+*_ of Li^+^ is in the order of 10^−11.5^–10^−13.1^ cm^2^ s^−1^, which is highly comparable to those well-designed metal oxide-based electrode materials (Zhao et al., 2020). CV curves were also tested to explore the reaction kinetics of the hierarchical WO_3_ agglomerate electrode. Figure 4C demonstrates the CV curves of the hierarchical WO_3_ agglomerate electrode at various sweep rates from 0.2 to 5 mV s^−1^ within a voltage range of 0.01–3 V. As the scan rates increase, the CV profiles remain similar except that the cathodic and anodic peaks slightly shift and gradually broaden. A power-law (*i* = a*v* ^b^) can be used to describe the relationship between the peak current (*i*) and scan rate (*v*), where *a* and *b* are defined as adjustable parameters (He et al., 2014; Liu et al., 2021). In principle, a *b* value of 0.5 means that the current is controlled by semi-infinite diffusion, while 1.0 indicates a surface capacitive-controlled behavior (Wang et al., 2019; Fu et al., 2020). By fitting the profiles of log(*i*) as a function of log(*v*) (Figure 4D), the calculated *b* values of 0.71 and 0.51 are computed for the cathodic and anodic peaks, respectively, indicating that the charge storage of the hierarchical WO_3_ agglomerates is mainly dominated by the joint effect of diffusion and capacitive-controlled processes (Hwang et al., 2019; Liang et al., 2021b).

**FIGURE 5:**
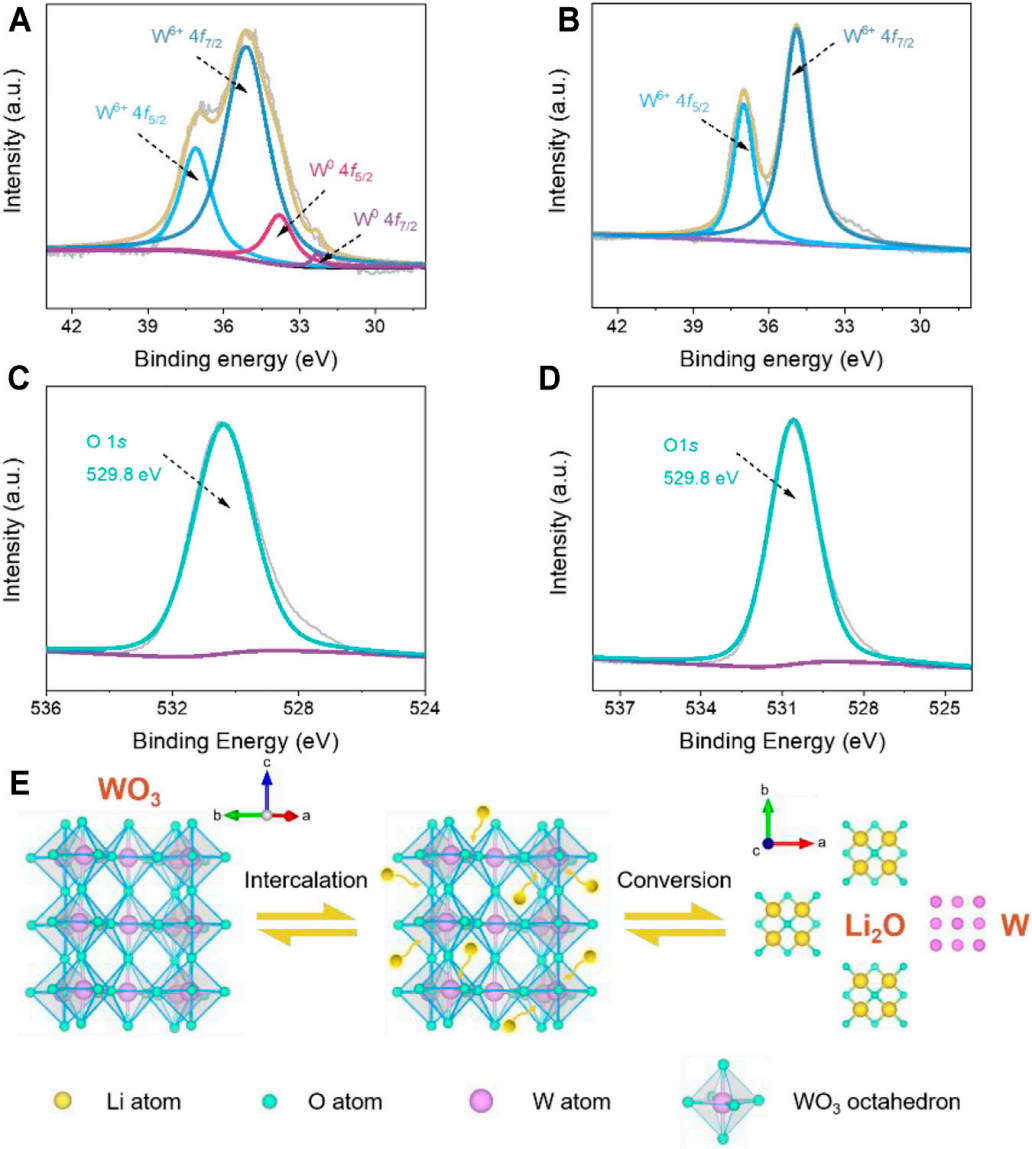
**(A, B)** *Ex situ* XPS spectra of the W 4*f* region of fully discharged and charged hierarchical WO_3_ agglomerate electrodes. **(C, D)** O 1*s* region of fully discharged and charged hierarchical WO_3_ agglomerate electrodes. **(E)** Schematic illustration of the proposed charge storage mechanism for WO_3_-based LIBs.

To reveal the charge storage mechanism of the hierarchical WO_3_ agglomerates, the *ex situ* XPS analyses are conducted at fully discharged/charged states to explore the valence state of tungsten and oxygen elements. The high-resolution XPS of W 4*f* presented in Figure 5A shows that two peaks appeared at binding energies of 35.1 and 37.1 eV at the fully discharged state, which can be ascribed to the W4*f*_7/2_ and W4*f*_5/2_ of W^6+^, respectively (Tong et al., 2016). In addition, two other small W 4*f* core level peaks centered at 32.1 and 33.5 eV also emerge in the XPS spectra, indicative of the formation of elemental tungsten as a result of Li^+^ intercalation (Tong et al., 2016). After being fully charged (Figure 5B), the doublet at 34.9 and 37.1 eV are detected in the high-resolution XPS of W 4*f*, which corresponds to the W4*f*_7/2_ and W4*f*_5/2_ of W^6+^. Figures 5C,D show the O 1*s* XPS spectra of the hierarchical WO_3_ agglomerates at fully discharged and charged stages. As demonstrated, the XPS measurement shows a stable typical peak located at 529.8 eV, which is attributed to the W-O bonding mode of WO_3_ (Li P. et al., 2016), indicating that oxygen does not participate in charge compensation during cycling. According to previous works (Kumagai et al., 1996; Gu et al., 2007; Huang et al., 2008), the energy storage in WO_3_ has been proved to be highly associated with lithium intercalation/de-intercalation during the cycling process. Combining with the XPS results, we further confirm that the charge storage mechanism of the prepared hierarchical WO_3_ agglomerates firstly occurs with Li^+^ intercalation and then the conversion of partial tungsten elements during the discharging process, which can also be found in other metal oxides (Poizot et al., 2000; Grugeon et al., 2001).

Based on the above analysis and discussions, the reaction mechanism of the hierarchical WO_3_ agglomerates in the charge/discharge processes can be schematically illustrated in Figure 5E, and the proposed mechanisms can be approximately expressed as follows (Wu and Yao, 2017; Wang et al., 2019):

$$WO_{3}+xLi^{+}+xe^{-}↔Li_{x}WO_{3}$$ (2)

$$Li_{x}WO_{3}+\left(6-x\right)Li^{+}+\left(6-x\right)e^{-}↔W+3Li_{2}O$$ (3)

The Li^+^ insertion and extraction reaction are represented by Eq. 2, and x represents the ratio of lithium ion to tungsten oxide. Eq. 3 shows that the typical conversion reaction occurs at the electrode, where the zero-valent tungsten can be generated. The mechanistic insights into the energy storage processes in WO_3_-based materials are helpful to design and prepare advanced electrode structures in enhancing the Li storage performances for high-energy LIBs.

## Conclusion

To summarize, hierarchical WO_3_ agglomerates assembled with straight and parallel aligned nanoribbons have been successfully prepared by a facile hydrothermal approach, and introduced as a novel promising anode material for LIBs. The unique hierarchical agglomerate structure building by secondary 1D nanoribbons with a large interior space can effectively increase the electrode/electrolyte contact area, provide sufficient accessible active sites, and enable rapid transport of both Li ions and electrons. Electrochemical tests demonstrate that the prepared hierarchical WO_3_ agglomerates show remarkable Li storage properties including high reversible specific capacity, outstanding rate capability, and excellent cycling stability, making it a very attractive anode for LIBs. This study paves the way to develop transition-metal-based electrode materials with well-designed architecture for applications in high-performance LIBs.

## Data Availability

The original contributions presented in the study are included in the article/Supplementary Material. Further inquiries can be directed to the corresponding authors.
