# Supplementary material for: Architecting Hierarchical WO3 Agglomerates Assembled With Straight and Parallel Aligned Nanoribbons Enabling High Capacity and Robust Stability of Lithium Storage
Source: Front Chem. 2022 Feb 2;9:834418. doi: 10.3389/fchem.2021.834418 (PMC8847682; doi:10.3389/fchem.2021.834418)
Supplement: Supplementary file 1 [file DataSheet1.docx]

Supplementary Material

# Supplementary Figures

**Supplementary Figure 1.** Survey XPS spectra of the hierarchical WO_3_ agglomerates electrodes.


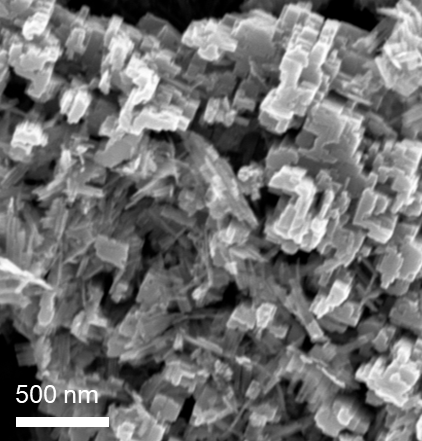


**Supplementary Figure 2.** FESEM image of the prepared WO_3_ bricks.

**
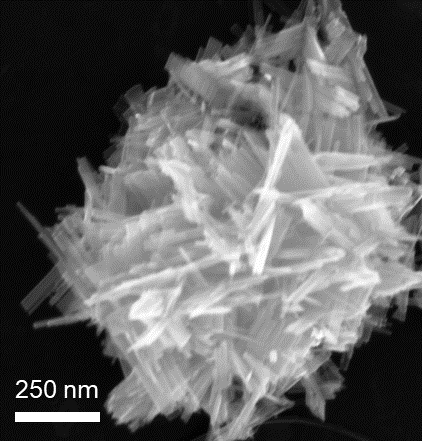
**

**Supplementary Figure 3.** FESEM image of the prepared WO_3_ micro-spheres.

**Supplementary Figure 4.** XRD patterns of WO_3_ bricks.

**Supplementary Figure 5.** XRD patterns of WO_3_ micro-spheres.


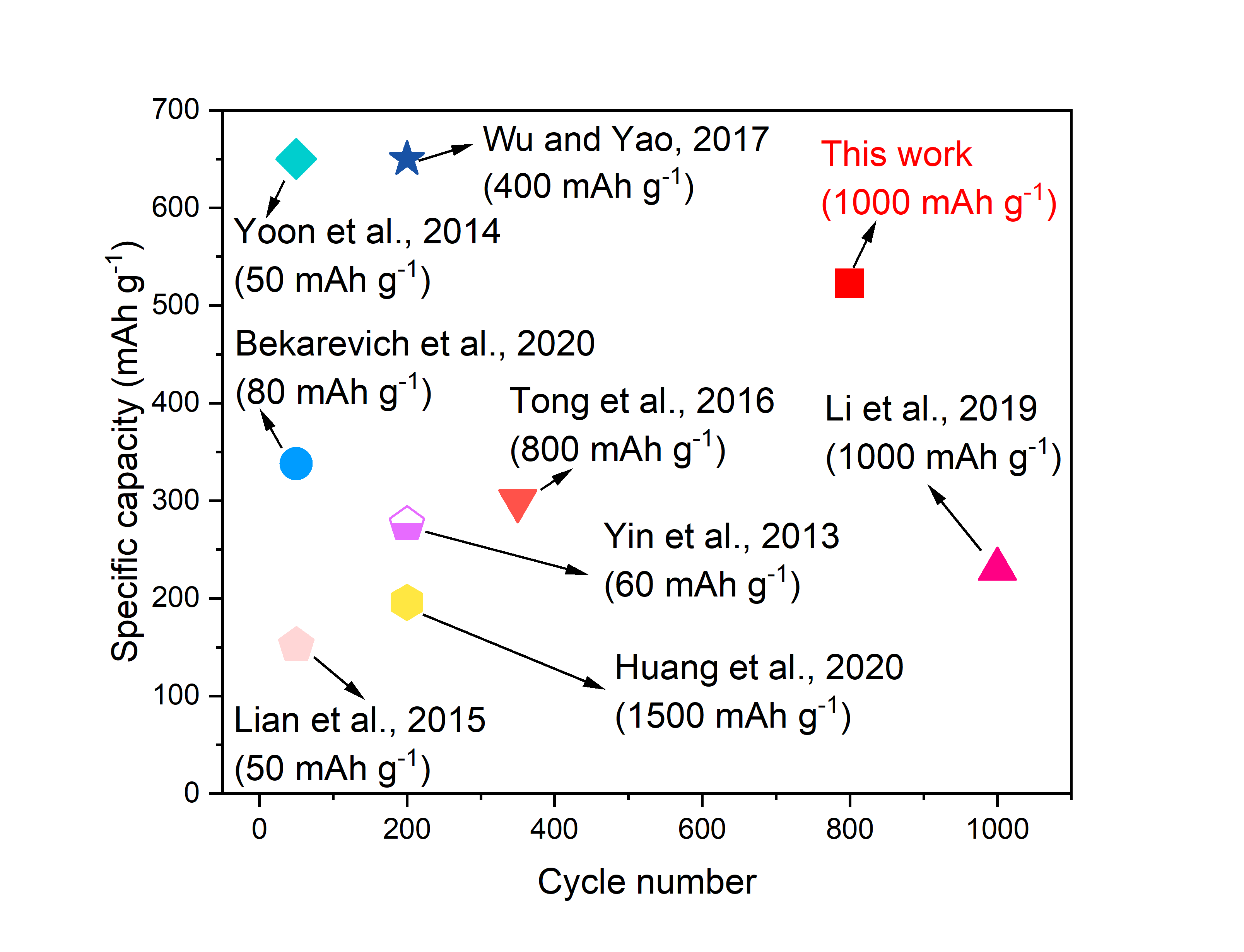


**Supplementary Figure 6.** Ragone plots for metal oxides electrode materials in LIBs.

# Supplementary Tables

**Supplementary Table 1.** Comparative electrochemical performance of hierarchical WO_3_ agglomerates with other works for LIBs reported.

| Cathode | Discharge capacity (mAh g^-1^) | Cycle number | Current density (mA g^-1^) | Ref. |
| --- | --- | --- | --- | --- |
| **hierarchical WO_3_ agglomerates** | **522.7** | **800** | **1000** | **This work** |
| WO_3_-NRs | 338 | 50 | 80 | (Bekarevich et al., 2020) |
| 20 wt% WO_3-x_ nanosheet | 230 | 1000 | 1000 | (Li et al., 2019) |
| WO_3_ hollow microspheres | 300 | 350 | 800 | (Tong et al., 2016) |
| cauliflower-like carbon-coated WO_3_ | 650 | 50 | 50 | (Yoon et al., 2014) |
| h-WO_3_/NSG | 196 | 200 | 1500 | (Huang et al., 2020) |
| WO_3_ nanotube bundle | 650 | 200 | 400 | (Wu and Yao, 2017) |
| WO_3_ nano-ribbons | 152 | 50 | 50 | (Lian et al., 2015) |
| γ- WO_3_ hierarchical  nanostructure | 276 | 200 | 60 | (Yin et al., 2013) |

**References**

Bekarevich, R., Pihosh, Y., Tanaka, Y., Nishikawa, K., Matsushita, Y., Hiroto, T., et al. (2020). Conversion Reaction in the Binder-Free Anode for Fast-Charging Li-Ion Batteries Based on WO_3_ Nanorods. *ACS Applied Energy Materials* 3(7)**,** 6700-6708. doi: 10.1021/acsaem.0c00844.

Huang, Y., Lu, R., Wang, M., Sakamoto, J., and Poudeu, P.F. (2020). Hexagonal-WO_3_ nanorods encapsulated in nitrogen and sulfur co-doped reduced graphene oxide as a high-performance anode material for lithium ion batteries. *Journal of Solid State Chemistry* 282**,** 121068. doi: 10.1016/j.jssc.2019.121068

Li, Y., Chang, K., Tang, H., Li, B., Qin, Y., Hou, Y., et al. (2019). Preparation of oxygen-deficient WO_3_- nanosheets and their characterization as anode materials for high-performance Li-ion batteries. *Electrochimica Acta* 298**,** 640-649. doi: 10.1016/j.electacta.2018.12.137.

Lian, C., Xiao, X., Chen, Z., Liu, Y., Zhao, E., Wang, D., et al. (2015). Preparation of hexagonal ultrathin WO_3_ nano-ribbons and their electrochemical performance as an anode material in lithium ion batteries. *Nano Research* 9(2)**,** 435-441. doi: 10.1007/s12274-015-0924-6.

Tong, H., Xu, Y., Cheng, X., Zhang, X., Gao, S., Zhao, H., et al. (2016). One-pot solvothermal synthesis of hierarchical WO_3_ hollow microspheres with superior lithium ion battery anode performance. *Electrochimica Acta* 210**,** 147-154. doi: 10.1016/j.electacta.2016.05.154.

Wu, X., and Yao, S. (2017). Flexible electrode materials based on WO_3_ nanotube bundles for high performance energy storage devices. *Nano Energy* 42**,** 143-150. doi: 10.1016/j.nanoen.2017.10.058.

Yin, J., Cao, H., Zhang, J., Qu, M., and Zhou, Z. (2013). Synthesis and Applications of γ-Tungsten Oxide Hierarchical Nanostructures. *Crystal Growth & Design* 13(2)**,** 759-769. doi: 10.1021/cg301469u.

Yoon, S., Woo, S.-G., Jung, K.-N., and Song, H. (2014). Conductive surface modification of cauliflower-like WO_3_ and its electrochemical properties for lithium-ion batteries. *Journal of Alloys and Compounds* 613**,** 187-192. doi: 10.1016/j.jallcom.2014.06.010.
